# Influence of infection control for COVID-19 on nutrition in relatively healthy Japanese HD patients: a retrospective observational study

**DOI:** 10.1007/s10157-025-02638-3

**Published:** 2025-02-28

**Authors:** Yuki Chiba, Ryotaro Takahashi, Rui Makino, Mai Yoshida, Koji Okamoto, Tasuku Nagasawa, Ichiro Kato, Sadatoshi Ito, Tetsuhiro Tanaka, Mariko Miyazaki

**Affiliations:** 1 Department of Nephrology, Tohoku University Graduate School of Medicine, Sendai, Japan; 2 Kawadaira Medical Clinic, Sendai, Japan; 3 Sendai Tokusyukai Hospital, Sendai, Japan; 4 Katta General Hospital, Shiroishi, Japan

**Keywords:** Body composition, COVID-19, Hemodialysis, Nutrition

## Abstract

**Background:**

Infection control for the novel coronavirus disease 2019 (COVID-19) has been linked to decreased physical activity and nutritional deterioration in the general population; however, the influence on hemodialysis (HD) patients is not well discussed.

**Methods:**

This multicenter retrospective study utilized the Geriatric Nutritional Risk Index (GNRI), Survival Index, and Nutritional Risk Index for Japanese HD patients (NRI-JH) to assess nutritional status and body composition over five observation periods. The primary endpoint was the body fluid removal rate (%) pre- and post-HD, whereas secondary endpoints included changes in GNRI, SI, body composition, and differences in NRI-JH.

**Results:**

We enrolled 139 HD patients in three facilities. The results showed a decrease in GNRI score, which indicates nutritional deterioration, between February 2020 and August 2020 (96.8 (93.2–98.9) vs. 93.8 (90.8–97.6)) (*P* = 0.0005). Multivariable analysis revealed that nutritional deterioration was associated with higher C-reactive protein and lower hemoglobin levels (*P* = 0.0004 and *P* = 0.0010, respectively), which were more noticeable in the urban facility. Furthermore, nutritional deterioration was linked to a decrease in soft lean and somatic cell mass and an increase in body fat mass, suggesting reduced physical activity.

**Conclusions:**

Nutritional deterioration was observed shortly after the first COVID-19 outbreak, suggesting an association with decreased physical activity.

**Supplementary Information:**

The online version contains supplementary material available at 10.1007/s10157-025-02638-3.

## Introduction

The number of patients undergoing chronic hemodialysis (HD) in Japan is increasing, reaching approximately 340,000 in 2020, with 69.1% of them aged ≥ 65 years [1]. Undernutrition in HD patients is associated with multiple factors, such as inflammatory cytokines, uremic pathology, and dietary restrictions, suggesting an increased risk of cardiovascular disease (CVD) and mortality [2]. Approximately half of the deaths in Japanese HD patients result from heart failure and infectious diseases; thus, nutritional intervention is crucial [1]. The novel coronavirus disease 2019 (COVID-19), first documented in Wuhan City, China, in December 2019, rapidly spread worldwide. We implemented strict infection control measures, restricted outings, and avoided contact with others to prevent infection. The general population experienced a decline in physical activity and nutritional deterioration during the COVID-19 outbreak [3–5]; however, the impact on socially vulnerable HD patients has not been well documented. This study aimed to identify the impact on body composition and nutritional indicators in HD patients during the COVID-19 outbreak.

## Materials and methods

### Patients’ selection

This multicenter retrospective study was conducted in Miyagi Prefecture, Tohoku Region, Japan. Patients were enrolled at the following three facilities in Miyagi Prefecture. Kawadaira Medical Clinic is in Sendai City, the biggest city in Miyagi Prefecture, with a population of 1.09 million as of December 2022. Katta General Hospital is in Shiroishi City, where the rural areas have a population of 31 thousand as of December 2022. Minamisanriku Hospital is in Minamisanriku-cho, where the coastal areas have a population of 11 thousand as of December 2022.

Inclusion criteria were defined as follows: (1) initiation of HD up to July 2018 and receiving HD in April 2021; (2) age between 20 and 90 years, regardless of sex. Exclusion criteria were as follows: (1) initiation of HD in August 2018 or later; (2) undergoing peritoneal dialysis; (3) inpatients during the observation periods, except for ophthalmologic and vascular access surgeries with shorter hospitalization; (4) difficulty walking independently.

### Data collection

According to the annual survey of the Japanese Society for Dialysis Therapy (JSDT), the recent 2 and 5 year survival rates of Japanese HD patients were approximately 80% and 60%, respectively [6]. Taking into account the possibility of participant dropouts, the observation period was set at 2 years. In addition, seasonal variations in HD patients were uncovered [7]; thus, we divided 2 years into five observation periods. Using an electronic medical record survey, we evaluated clinical characteristics and pre-dialysis laboratory values. Because of the retrospective nature of the study, data from approximately 2 months before and after the observation periods were included.

### Endpoint

The primary endpoint was defined as the body fluid removal rate (%) between pre- and post-HD, on the day following a 2-day interval between HD sessions. In addition, we evaluated fluid removal. The fluid removal rate (%) and total fluid removal were calculated using the following equations:

$$\text{Fluid removal rate}\; (\%) = ((\text{Pre-HD body weight} \;(\text{Bw}) - {\text{Post-HD Bw}}) / {\text{Post-HD Bw}}) \times 100$$

$${\text{Fluid removal (kg) = Pre - HD Bw }} - {\text{Post - HD Bw}}$$

The secondary endpoints included the % Geriatric Nutritional Risk Index (GNRI), Survival Index (SI), body composition change, and the difference in the Nutritional Risk Index for Japanese HD patients (NRI-JH).

The % GNRI and body composition change were calculated using the following formula (e.g., February 2019 vs. August 2019):

$${\text{((Scores or values in August 2019 - Scores or values in February 2}}0{19})/{\text{Scores or values in February 2}}0{19}) \times 100(\%)$$

The difference in NRI-JH change was calculated using the following formula (e.g., February 2019 vs. August 2019):

(Scores in August 2019 – Scores in February 2019).

### Nutritional indicators

GNRI is an improved version of NRI used in the surgical field in 2005 for older patients [8]. The risk of mortality was high (GNRI: < 82), moderate (GNRI: 82 to < 92), and low (GNRI: 92 to ≤ 98) [5]. The GNRI score is calculated according to the following equations:

$$GNRI \, = \, (14.89 \times albumin (Alb) (g/dl)) + (41.7 \times current Bw / ideal Bw)$$

The BCG method overestimates Alb levels in HD patients, while the improved BCP method underestimates them [9]. If Alb levels by the improved BCP method are ≤ 3.5 g/dL, we converted the following formula (measured levels + 0.3), as per the recommendation for converting Alb concentration presented by the Japanese Society of Laboratory Medicine. The post-HD Bw was used as the current Bw. The ideal Bw was a body-mass index (BMI) of 22; thus, we defined the ideal Bw as the value of 22 × height (m)^2^. If the current Bw was greater than the ideal Bw in this formula, the current/ideal Bw was set to 1.0 [8].

The NRI-JH score was calculated according to BMI and serum Alb, creatinine (Cr), and total cholesterol (T-Chol), and are detailed in Table 1 [10, 11]. BMI was calculated using post-HD Bw, and this study used the pre-dialysis values of serum Alb, Cr, and T-Chol for NRI-JH. NRI-JH detects the risk of malnutrition on mortality in HD patients. Patients were categorized into two groups: low-risk (score 0–7) and high-risk (score 8–13) groups [10].

**Table 1:** Nutritional Risk Index for Japanese HD patients

Factors		Score
BMI*	< 20.0 kg/m^2^	3
	≥ 20.0 kg/m^2^	0
Serum Alb	BCG method** BCP method***	
Age < 65 years	< 3.7 g/dL < 3.4 g/dL	4
	≥ 3.7 g/dL ≥ 3.4 g/dL	0
Age ≥ 65 years	< 3.5 g/dL < 3.2 g/dL	4
	≥ 3.5 g/dL ≥ 3.2 g/dL	0
Serum Cr	Male Female	
Age < 65 years	< 11.6 mg/dL < 9.7 mg/dL	4
	≥ 11.6 mg/dL ≥ 9.7 mg/dL	0
Age ≥ 65 years	< 9.7 mg/dL < 8.0 mg/dL	4
	≥ 9.7 mg/dL ≥ 8.0 mg/dL	0
Serum T-Chol	≥ 220 mg/dL	2
	< 130 mg/dL	1
	130 ≤ T-Chol < 220 mg/dL	0
(From ref. 10)		

Pre-dialysis values after a 2-day interval between HD are used for these factors, *Alb* albumin, *BCG* bromocresol green, *BCP* bromocresol purple, *BMI* body mass index, *Cr* creatinine, *HD* hemodialysis, *T-Chol* total cholesterol

^*^Post-HD Bw was used for BMI calculation

^**^The BCG method was used at the Kawadaira Medical Clinic

^***^The BCP method was used at the Katta General Hospital and Minamisanriku Hospital

The SI was developed using data from HD patients in the Dialysis Outcomes and Practice Patterns Study (DOPPS), and a lower SI can detect higher-risk HD patients for all-cause mortality [12]. The SI is composed of age, BMI, serum Cr, serum Alb, serum T-Chol, serum inorganic phosphorus (IP), history of CVD, and the presence or absence of an arteriovenous fistula (AVF), and is calculated using the following equations [12]:

$$SI = 10 - \left( {0.4 \times Age\left( {year} \right)} \right) + \left( {0.3 \times BMI\left( {kg/m^{2} } \right)} \right) + \left( {0.7 \times Cr\left( {mg/dl} \right)} \right) + \left( {6 \times Alb\left( {g/dl} \right)} \right) + \left( {0.03 \times T-Chol\left( {mg/dl} \right)} \right) - \left( {IP\left( {mg/dl} \right)} \right) - \left( {2 \times CVD} \right) + \left( {2 \times AVF} \right)$$

Post-HD Bw was used for BMI calculation in this study.

CVD as a complication (yes = 1, no = 0).

AVF use (yes = 1, no = 0).

### Body composition

At the Kawadaira Medical Clinic, body composition was assessed with InBody770^Ⓡ^ (InBody Japan, Tokyo, Japan) using multi-frequency bio-electrical impedance analysis according to a general guide.

### Statistical analysis

Details on statistical analyses are provided in the Supplementary Methods.

## Results

### Clinical characteristics of patients

**Fig. 1:**
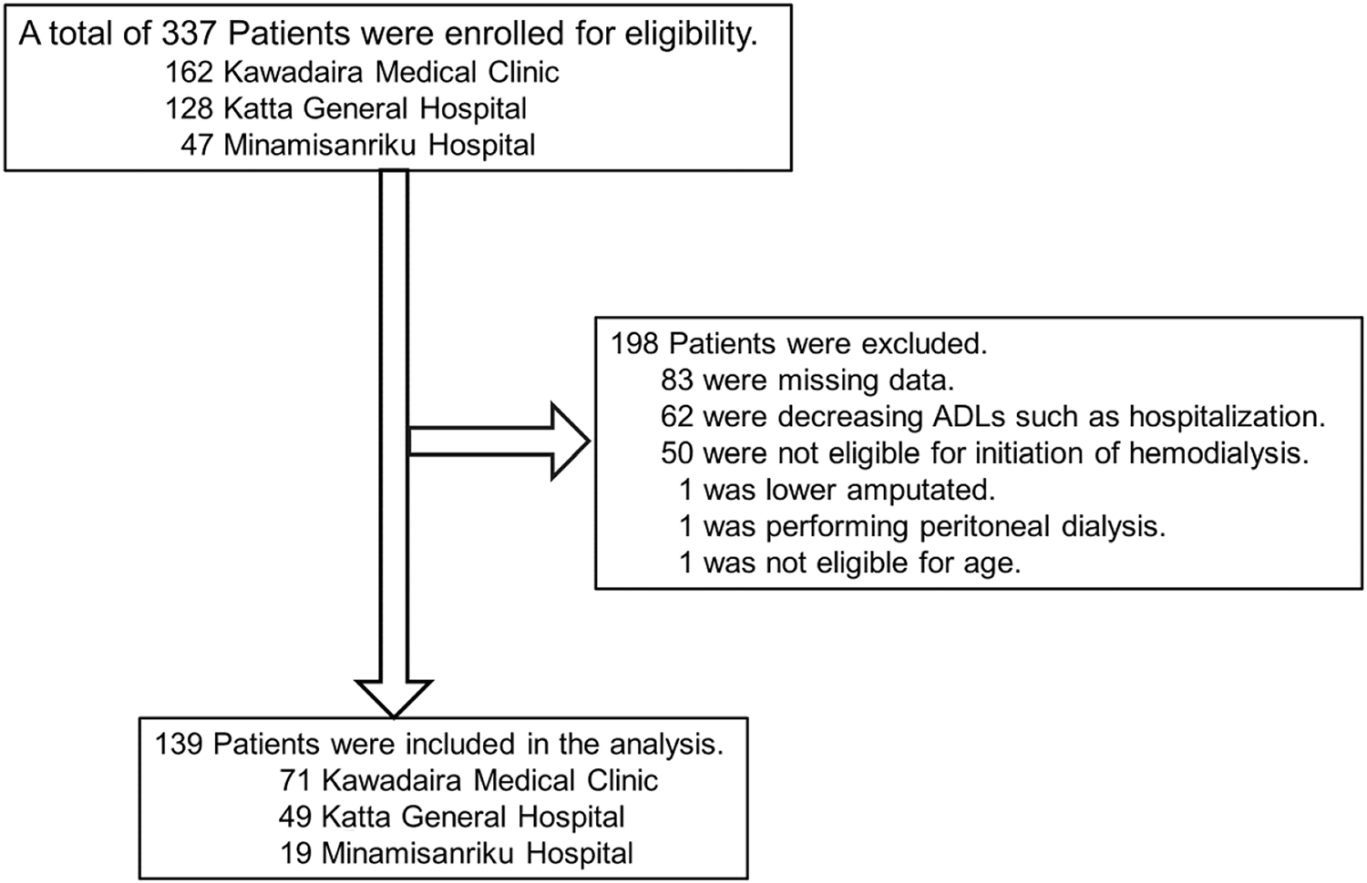
Flow chart of patients’ selection

**Fig. 2:**
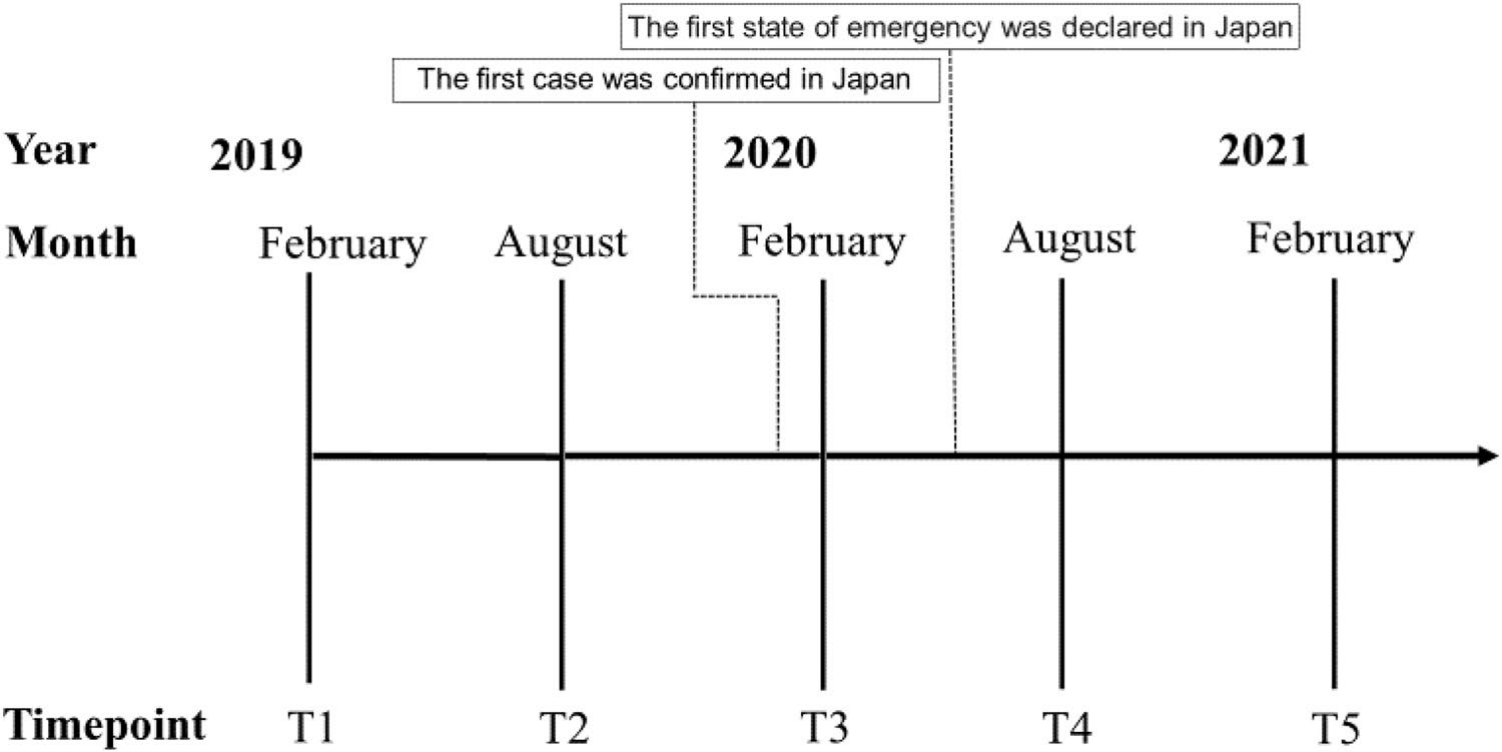
The five observation periods. Dates around 2 months before and after each period are allowed. The first case of COVID-19 occurred on January 16, 2020. The government declared the first state of emergency for COVID-19 in Japan, which applied to Tokyo and several of the most affected prefectures on April 7, 2020. The state was eventually expanded nationwide on April 16. *COVID*-19 novel coronavirus disease 2019

The patients’ selection flow diagram is shown in Fig. 1. A total of 337 patients were assessed for eligibility and finally 139 patients were included in these analyses. The five observation periods are shown in Fig. 2, and the clinical characteristics of patients in February 2021 are shown in Table 2. The median age was 68.3 (60.3–74.3) years, with males constituting 76.3%. Concerning HD therapy, the median duration was 9.2 (4.9–13.5) years and 77.7% of patients received daytime HD. The type of vascular access was arterio-venous fistulas (95.0%), and the remaining patients had arterio-venous grafts. The laboratory values of enrolled patients in observation periods are shown in Table 3. The results of the ANOVA revealed an obvious difference in only serum Alb concentration between observation periods (*P* < 0.0001). The Alb levels in August 2020 are lower than those in February 2019, August 2019, and February 2020 statistically significant *(P* = 0.0013, *P* = 0.0013, and *P* < 0.0001, respectively).

**Table 2:** Patients’ characteristics and contents of HD in February 2021

Variable	Results (*n* = 139)
Age (year)	68.3 (60.3–74.3)
Male sex	106 (76.3)
Duration of HD therapy (year)	9.2 (4.9–13.5)
Time of HD (hour)	4.2 ± 0.4
Daytime HD	108 (77.7)
Type of vascular access	AVF 132 (95.0), AVG 7
Primary disease of ESKD	
Diabetic mellitus	52 (37.4)
Chronic glomerulonephritis	39 (28.1)
Nephrosclerosis	20 (14.4)
Polycystic kidney disease	6 (4.3)
Vesicoureteral reflux	5 (3.6)
Others	17 (12.2)

Values are presented as means ± standard deviation, median (interquartile range), or the number of patients (%)

*AVF* arteriovenous fistulas, *AVG* arteriovenous graft, *HD* hemodialysis, *ESKD* end-stage kidney disease

**Table 3:** Blood test results across observation periods

Variable	February 2019	August 2019	February 2020	August 2020	February 2021	*P* value
Hb (g/dL)	11.1 (10.3–11.8)	11.0 (10.3–11.8)	11.2 (10.5–11.9)	11.1 (10.5–11.8)	11.2 (10.5–11.8)	0.80
K (mEq/L)	4.8 (4.3–5.3)	4.7 (4.3–5.0)	4.7 (4.3–5.2)	4.6 (4.2–5.2)	4.7 (4.3–5.2)	0.39
Ca (mg/dL) *	9.2 ± 0.7	9.2 ± 0.6	9.3 ± 0.7	9.4 ± 0.7	9.3 ± 0.6	0.08
IP (mg/dL)	5.3 (4.3–6.2)	5.0 (4.2–6.0)	5.0 (4.3–5.9)	5.0 (4.2–5.9)	5.2 (4.4–6.1)	0.28
Mg (mg/dL) **	2.6 ± 0.4	2.5 ± 0.4	2.5 ± 0.4	2.4 ± 0.4	2.5 ± 0.3	0.15
BUN (mg/dL)	59.4 ± 14.5	58.5 ± 13.8	58.7 ± 14.3	56.0 ± 13.8	58.5 ± 14.1	0.33
Cr (mg/dL)	10.4 (8.9–11.8)	10.9 (9.0–12.3)	10.2 (8.9–12.1)	10.3 (8.9–12.1)	10.0 (8.9–11.7)	0.40
CRP (mg/dL)	0.12 (0.05–0.32)	0.11 (0.05–0.34)	0.13 (0.06–0.40)	0.11 (0.05–0.34)	0.13 (0.07–0.31)	0.60
Alb (g/dL) ***	3.7 (3.6–3.9)	3.7 (3.6–3.9)	3.8 (3.6–3.9)	3.6 (3.5–3.8)	3.7 (3.5–3.8)	** < 0.0001**
T-Chol (mg/dL)	164.0 (142.0–190.0)	159.0 (135.0–180.0)	160.0 (140.0–192.0)	160.0 (137.0–186.0)	160.0 (142.0–186.0)	0.62

Values are presented as means ± standard deviation or median (interquartile range). Pre-dialysis values after a 2-day interval between HD are indicated for blood tests. *P*-value resulting from variance analysis (ANOVA) and bold values indicate statistical significance

*Alb* albumin, *BUN* blood urea nitrogen, *Ca* calcium, *Cr* creatinine, *CRP* C-reactive protein, *Hb* hemoglobin, *IP* inorganic phosphorus, *K* potassium, *Mg* magnesium, *T-Chol* total cholesterol

^*^Corrected values are used

^**^Missing dates are present

^***^Calculated values by the improved BCP method were converted

## Primary endpoint

Table 4 shows post-HD Bw (kg), BMI (kg/m^2^), fluid removal (kg), and fluid removal rate (%) in the observation periods. No significant differences in any endpoints were observed in each observation period.

**Table 4:** Values of weight, nutrition, and in-body testing in observation periods

Objective	February 2019	August 2019	February 2020	August 2020	February 2021	*P* value
Weight						
Post-HD Bw	62.4 (54.1–70.6)	62.8 (53.9–70.5)	62.3 (54.5–70.9)	61.9 (54.5–70.0)	62.4 (53.7–68.9)	1.00
BMI*	23.6 (20.6–26.1)	23.5 (20.8–25.9)	23.7 (20.9–26.2)	23.5 (20.6–26.1)	23.3 (20.7–25.8)	1.00
Fluid removal (kg)**	2.9 (2.4–3.5)	2.7 (2.2–3.4)	2.9 (2.3–3.5)	2.7 (2.2–3.4)	2.8 (2.3–3.5)	0.20
Fluid removal rate (%)***	4.7 (3.9–5.5)	4.3 (3.6–5.2)	4.6 (4.0–5.5)	4.4 (3.7–5.3)	4.7 (3.8–5.6)	0.13
Nutrition						
GNRI	96.6 (93.1–98.3)	95.9 (92.7–98.3)	96.8 (93.2–98.9)	93.8 (90.8–97.6)	95.3 (92.3–96.8)	** < 0.0001**
NRI-JH	4.0 (0.0–4.0)	3.0 (0.0–4.0)	4.0 (0.0–5.0)	4.0 (0.0–5.0)	4.0 (0.0–5.0)	0.29
SI	20.0 (15.7–25.5)	20.2 (15.6–25.3)	20.2 (15.8–25.5)	19.0 (14.7–23.2)	17.9 (14.6–24.0)	0.21
In-body						
SLM (kg)	45.4 ± 9.7	45.2 ± 9.6	45.7 ± 9.7	45.1 ± 9.3	45.3 ± 9.8	1.00
SMI (kg/m^2^)	7.3 ± 1.4	7.3 ± 1.4	7.3 ± 1.4	7.2 ± 1.4	7.2 ± 1.5	0.99
BFM (kg)	17.0 (12.9–23.5)	17.5 (12.9–24.8)	18.0 (11.4–23.4)	18.1 (10.9–25.7)	16.8 (11.7–24.5)	0.98
BCM (kg)	30.6 ± 6.4	30.6 ± 6.5	30.8 ± 6.5	30.5 ± 6.2	30.7 ± 6.6	1.00
PA (°)	4.4 ± 0.7	4.4 ± 0.7	4.4 ± 0.7)	4.3 ± 0.7	4.3 ± 0.7	0.88

Values are presented as means ± standard deviation or median (interquartile range)

Pre-dialysis values after a 2-day interval between HD are used for nutritional indicators

*P*-value resulting from variance analysis (ANOVA) and bold values indicate statistical significance

*BCM* body cell mass, *BFM* body fat mass, *BMI* body mass index, *Bw* body weight, *GNRI* geriatric nutritional risk index, *NRI-JH* nutritional risk index for Japanese HD patients, *HD* hemodialysis, *PA* phase angle, *SI* survival index, *SLM* soft lean mass, *SMI* skeletal muscle index

^*^Post-HD Bw after a 2-day interval between HD is used for calculating BMI

^**^Pre-HD Bw − Post-HD Bw

^***^((Pre-HD Bw − Post-HD Bw)/Post-HD Bw) × 100

## Secondary endpoints

**Fig. 3:**
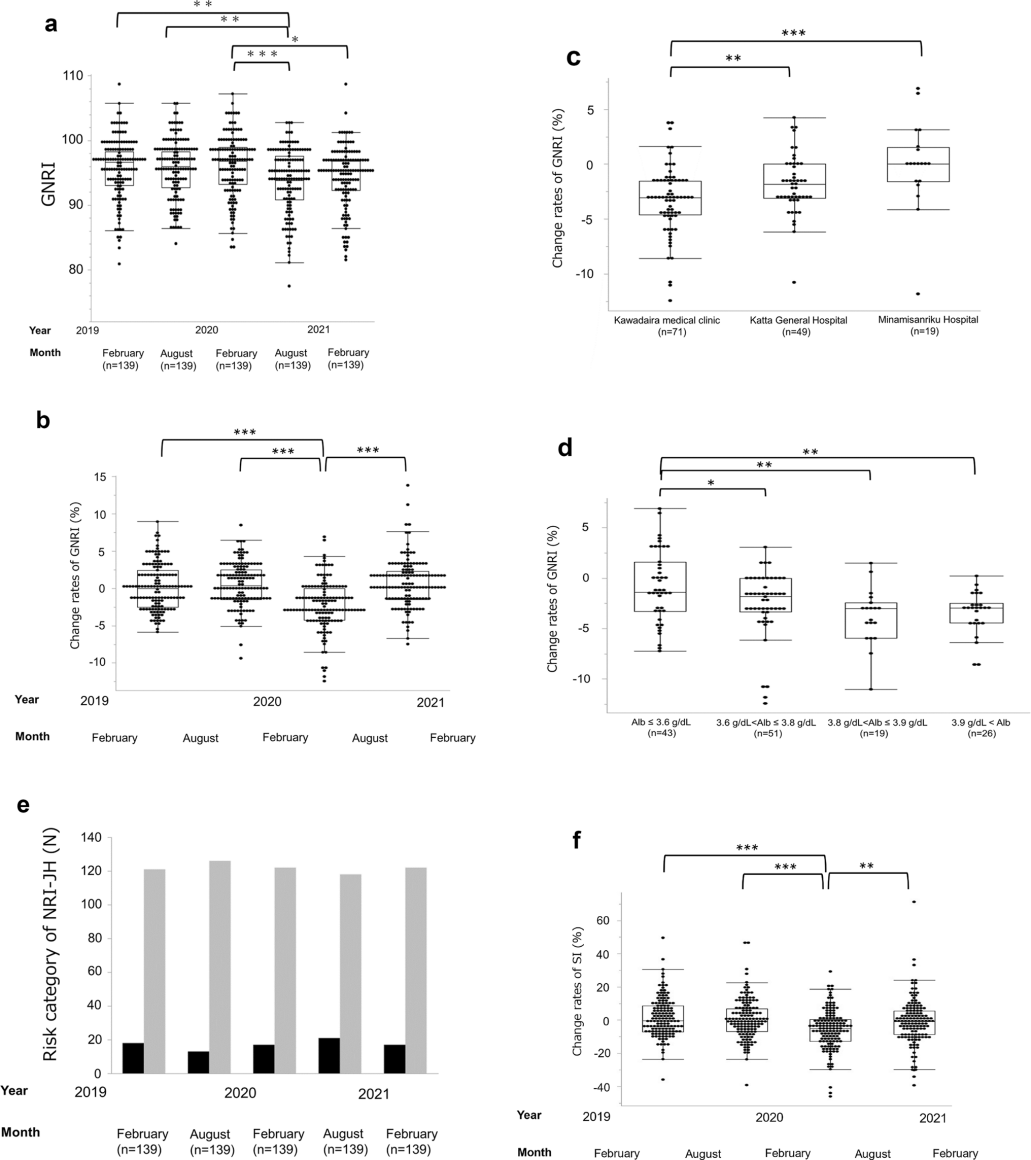
The nutritional status in observation periods and GNRI change (%) between observation periods. **a** GNRI scores in August 2020 are lower than those in February 2020, August 2019, and February 2019 and are statistically significant, suggesting nutritional deterioration immediately after the COVID− 19 outbreak. GNRI scores increased in February 2021 but were lower than those in February 2020. **b** GNRI shows an obvious decreasing trend from February 2020 to 6 months later, and no clear changes in other observation periods are observed. **c** GNRI change (%) from February 2020 to 6 months later declined most in patients at the Kawadaira Medical Clinic compared to other facilities. **d** GNRI change (%) from February 2020 to 6 months later declined most in patients in the low Alb level group (Alb ≤ 3.6 g/dL) compared to the other three groups. **e** Gray bar indicates the number of low-risk group patients (score 0–7), and the black bar shows the number of medium and high-risk group patients (score 8–13) in the risk category of NRI− JH. No clear differences in the risk category ratio of NRI− JH are observed. **f**: SI shows an obvious decreasing trend from February 2020 to 6 months later, and no clear changes in other observation periods are observed. *GNRI* geriatric nutritional risk index, *NRI*− *JH* nutritional risk index for Japanese HD patients, *SI* survival index. * *P* < 0.05, ** *P* < 0.01, *** *P* < 0.001

The GNRI scores in August 2020 were significantly lower than those in February 2019, August 2019, and February 2020 (93.8 (90.8–97.6) vs. 96.6 (93.1–98.3) vs. 95.9 (92.7–98.3) vs. 96.8 (93.2–98.9); *P* = 0.0075, *P* = 0.0077, and *P* = 0.0005, respectively) (Table 4) (Fig. 3a). A significant decrease in GNRI change (%) was observed between August 2019 and 6 months later, compared to the period from February 2020 to 6 months later (0.37 [− 1.52 to 2.46] vs. − 2.86 [− 4.24 to 0.0]). In addition, a significant increase in GNRI change (%) was noted between February 2020 to 6 months later and August 2020 to 6 months later (− 2.86 [− 4.24 to 0.0] vs. 0.25 [− 1.52 to 2.30]; *P* < 0.0001, respectively) (Table 5) (Fig. 3b). The results showed that nutritional status worsened shortly after the COVID-19 outbreak, from February 2020 to 6 months later, and recovery trends were observed from August 2020 to 6 months later. The decline in GNRI rates (%) from February 2020 to 6 months later was more pronounced in patients at the Kawadaira Medical Clinic compared to those at the Katta General Hospital and Minamisanriku Hospital (− 3.04 [− 4.62 to − 1.52] vs. − 1.84 [− 3.08 to 0.0] vs. 0.0 [− 1.56 to 1.54]; *P* = 0.0073 and *P* = 0.0002, respectively) (Fig. 3c). To evaluate the relationship between serum Alb concentration in February 2020 and GNRI change (%) from February 2020 to 6 months later, we divided the enrolled patients into four groups by quartile: Group 1 (Alb ≤ 3.6 g/dL), Group 2 (3.6 g/dL < Alb ≤ 3.8 g/dL), Group 3 (3.8 g/dL < Alb ≤ 3.9 g/dL) and Group 4 (3.9 g/dL < Alb) (Fig. 3d). The decline in GNRI rates (%) in Group 1 patients (Alb ≤ 3.6 g/dL) were less than those in Group 2–4 patients (− 1.37 [− 3.28 to 1.61] vs. − 1.84 [− 3.36 to 0.0] vs. − 2.98 [− 5.97 to − 2.44] vs. − 2.94 [− 4.45 to − 2.48]; *P* = 0.045, *P* = 0.0040, and *P* = 0.0051, respectively).

**Table 5:** GNRI and body composition change (%), and the difference in change in NRI-JH between observation periods

Objective	February 2019 to 6 months later	August 2019 to 6 months later	February 2020 to 6 months later	August 2020 to 6 months later	*P* value
Nutrition					
GNRI	0.0 (− 2.51–2.42)	0.37 (− 1.52–2.46)	− 2.86 (− 4.24–0.0)	0.25 (− 1.52–2.30)	** < 0.0001**
NRI− JH	0.0 (0.0–0.0)	0.0 (0.0–0.0)	0.0 (0.0–0.0)	0.0 (0.0–0.0)	**0.046**
SI	− 0.46 (− 7.06–8.67)	− 0.53 (− 6.82–6.77)	− 4.95 (− 12.7–0.34)	− 0.9 (− 8.71–5.60)	** < 0.0001**
InBody testing					
SLM	− 0.55 (− 3.65–2.02)	1.72 (− 1.30–3.38)	− 0.26 (− 3.58–1.09)	0.43 (− 2.04–3.46)	**0.0036**
SMI	0.0 (− 2.94–2.99)	0.0 (− 3.75–2.74)	0.0 (− 4.29–2.82)	0.0 (− 2.78–2.63)	0.42
BFM	3.20 (− 4.81–10.9)	− 3.65 (− 10.9–2.05)	0.28 (− 6.03–10.3)	− 2.50 (− 8.73–4.89)	**0.012**
BCM	0.0 (− 1.84–1.94)	0.91 (− 1.54–3.50)	− 0.99 (− 3.25–1.53)	0.78 (− 2.05–2.93)	**0.016**
PA	− 2.0 (− 7.27–4.35)	0.0 (− 4.35–5.88)	0.0 (− 6.38–5.77)	0.0 (− 7.14–5.13)	0.76

Values are presented as means ± standard deviation or median (interquartile range)

Calculate change rates (%) using the following formula (for example: between February 2019 and August 2019); ((Values in August 2019 – Values in February 2019)/Values in February 2019) × 100. Calculate the difference in change using the following formula (for example: between February 2019 and August 2019); (Scores in August 2019 – Scores in February 2019)

*P*-value resulting from variance analysis (ANOVA) and bold values indicate statistical significance

*BCM* body cell mass, *BFM* body fat mass, *GNRI* geriatric nutritional risk, *NRI-JH* nutritional risk index for Japanese HD patients, *PA* phase angle, *SI* survival index, *SLM* soft lean mass, *SMI* skeletal muscle index

On the other hand, no clear differences in the scores and risk classification ratio of NRI-JH were observed (Fig. 3e) (Table 4). The results of ANOVA revealed an obvious difference in the NRI-JH change (*P* = 0.046); however, no clear differences were observed between observation periods (Table 5).

The SI scores from the observation periods are shown in Table 4. No clear differences were observed in the SI scores between each period. A significant decrease in SI change (%) was observed between August 2019 to 6 months later and February 2020 to 6 months later (− 0.53 [− 6.82 to 6.77) vs. − 4.95 [− 12.7 to 0.34]) and significant increase between February 2020 to 6 months later and August 2020 to 6 months later (− 4.95 [− 12.7 to 0.34] vs. − 0.9 [− 8.71 to 5.60]; *P* < 0.001and *P* < 0.01, respectively) (Table 5) (Fig. 3f).

**Fig. 4:**
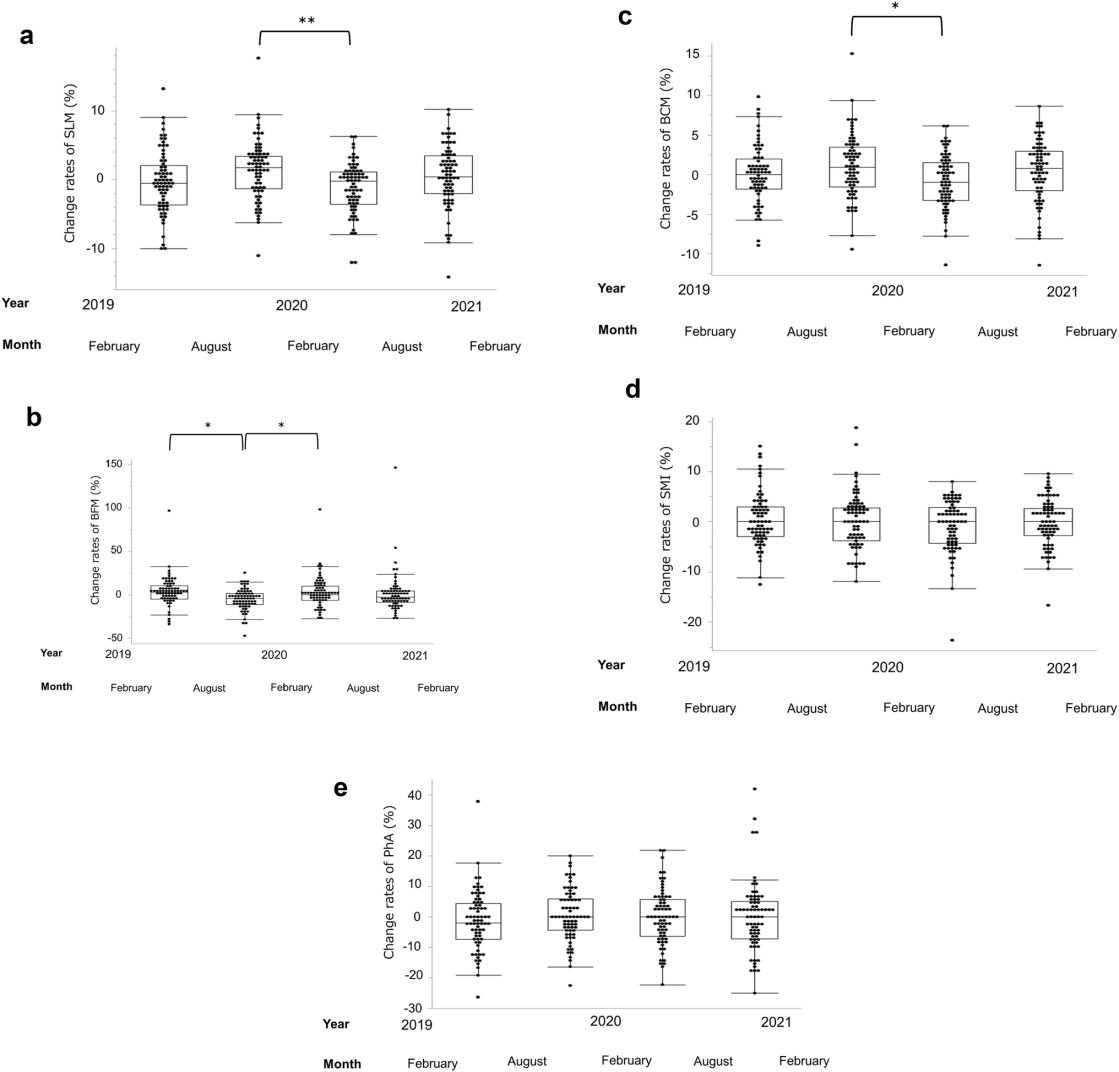
The body composition change (%) between observation periods in one facility. **a** SLM shows a decreasing trend from February 2020 to 6 months later, and the same period in 2019 also shows a decreasing trend. **b** BFM shows an increasing trend from February 2020 to 6 months later, and the same period in 2019 also shows an increasing trend. **c** BCM shows a decreasing trend from February 2020 to 6 months later, and the same period in 2019 also shows a decreasing trend. **d** No clear changes in SMI throughout the observation period are observed. **e** No clear changes in PhA throughout the observation period are observed. *BCM* body cell mass, *BFM* body fat mass, *COVID*-19 coronavirus disease 2019, *SLM* Soft lean mass. * *P* < 0.05, ** *P* < 0.01

Regarding body composition, a significant decrease in soft lean mass (SLM) change (%) was observed between August 2019 to 6 months later and February 2020 to 6 months later (1.72 [− 1.30 to 3.38] vs. − 0.26 [− 3.58 to 1.09]; *P* = 0.0029), as well as in body cell mass change (%) (0.91 [− 1.54 to 3.50] vs. − 0.99 [− 3.25 to 1.53]; *P* = 0.016) (Fig. 4a, c, respectively). A significant increase BFM change (%) was observed between August 2019 to 6 months later and February 2020 to 6 months later (− 3.65 [− 10.9 to 2.05] vs. 0.28 [− 6.03 to 10.3]; *P* = 0.045) (Fig. 4b).

**Fig. 5:**
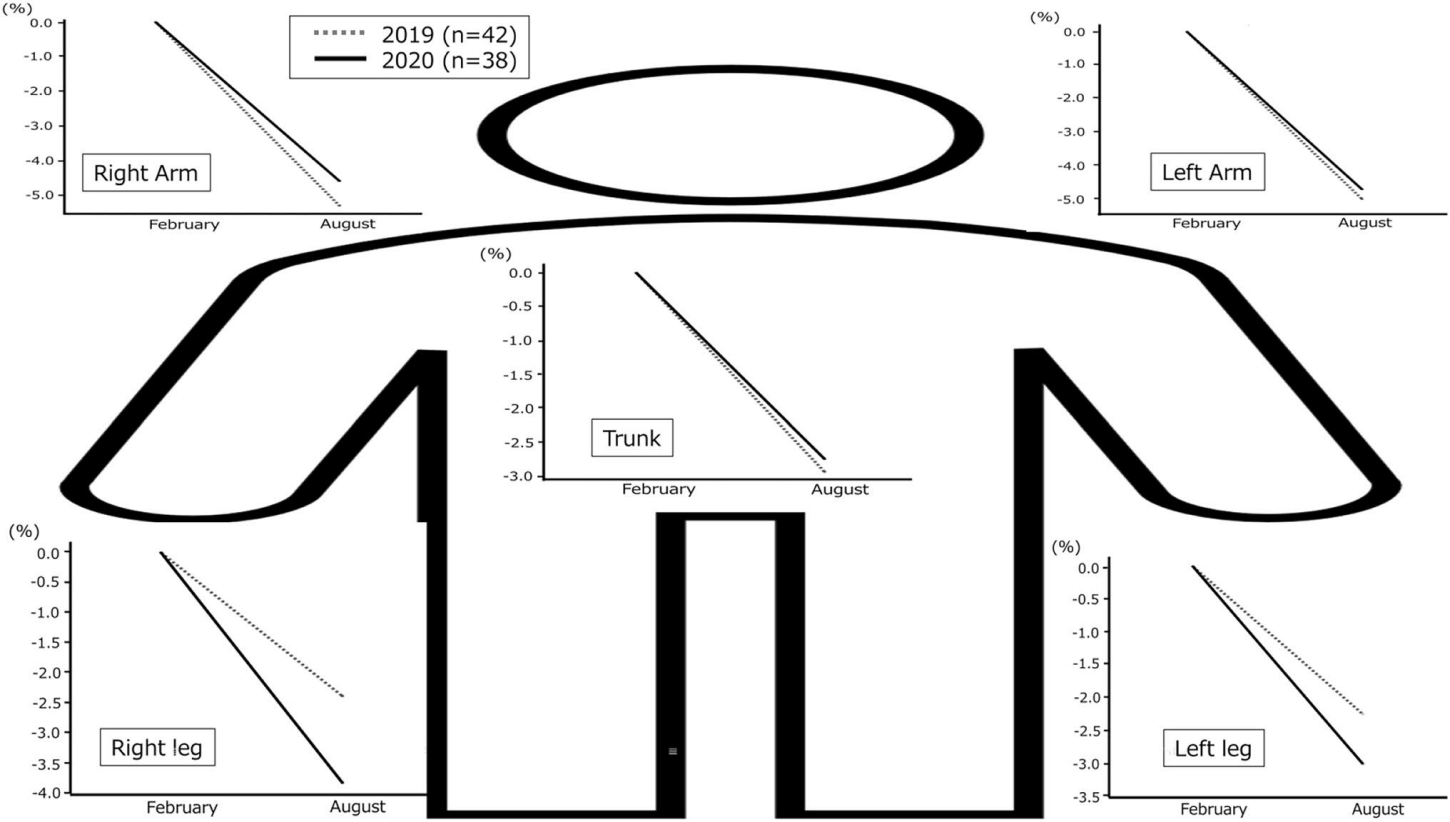
The regression line summarizes the percentage (%) of segmental SLM change from February to August 2019 and 2020. The dotted line represents the change (%) in 2019, and the black solid line represents those in 2020. From the left are February and August on the x-axis, and the y-axis shows the segmental SLM change (%). Clockwise from the top left are the right arm, left arm, left leg, and right leg change (%). In addition, the center graph represents the trunk change (%). Both 2019 and 2020 show that the decreasing rates of SLM in the upper limbs are greater; however, the decreasing rates of SLM in the lower limb in 2020 are greater than those in 2019. *SLM* soft lean mass

Decreasing trends in SLM were observed from February 2019 to 6 months later and February 2020 to 6 months later, with the segmental SLM change (%) showing a decline in patients during both periods, as shown in Fig. 5. Both periods showed that the decreasing rates of SLM in the upper limbs were greater; however, the decreasing rates in the lower limbs in 2020 were greater than those in 2019.

## Associated variables regarding the change rates of GNRI

Univariable and multivariable regression analyses associated with the GNRI change (%) from February 2020 to 6 months later are shown in Table 6. The results showed that hemoglobin (Hb) levels (g/dL) were positively associated with GNRI change (%) from February 2020 to 6 months later in univariable analysis (*P* = 0.014). In addition, the multivariable analysis demonstrated that Hb levels had a positive association (*P* = 0.0010), whereas C-reactive protein (CRP) levels were negatively associated (*P* = 0.0004), with stronger associations observed in the urban facility (*P* = 0.027).

**Table 6:** Univariable and multivariable regression analysis for the GNRI change (%) from February 2020 to 6 months later

Variable	Univariable analysis			Multivariable analysis		
	F	*γ*	*P* value	SE	β	*P* value
Age (years), < 70 or ≥ 70	0.087		0.77			
Sex, Female	1.12		0.29	0.29	0.11	0.16
Duration of HD therapy (year)	0.65	− 0.069	0.42			
Time of HD (h), < 4.5	2.26		0.14	0.30	0.079	0.30
HD, Daytime	3.24		0.074	0.31	0.11	0.18
BMI*	2.06	0.12	0.15			
Facility						
Kawadaira Medical Clinic	14.04		**0.0003**	0.29	− 0.20	**0.027**
Katta General Hospital	1.84		0.18			
Blood test*						
Hb	6.16	0.21	**0.014**	0.24	0.26	**0.0010**
K	0.021	0.012	0.89			
Ca (corrected)	7.95	− 0.23	**0.0055**	0.41	− 0.14	0.093
IP	2.39	− 0.13	0.12	0.20	0.078	0.33
BUN	1.78	− 0.11	0.18	0.020	0.046	0.58
Cr	0.028	− 0.014	0.87			
CRP	18.8	− 0.35	** < 0.0001**	0.24	− 0.28	**0.0004**
Alb	30.7	0.43	** < 0.0001**			
T− Chol	0.027	0.014	0.87			
InBody testing*						
SLM	0.00	− 0.00038	1.00			
SMI	0.039	− 0.024	0.84			
BFM	0.22	0.056	0.64			
BCM	0.0023	0.0058	0.96			
PA	0.24	0.058	0.63			

Bold values indicate statistical significance. *Alb* albumin, *BCM* body cell mass, *BFM* body fat mass, *BMI* body mass index, *BUN* blood urea nitrogen, *Ca* calcium, *Cr* creatinine, *CRP* C-reactive protein, *GNRI* geriatric nutritional risk index, *Hb* hemoglobin, *HD* hemodialysis, *IP* inorganic phosphorus, *K* potassium, *Mg* magnesium, *PA* phase angle, *SLM* soft lean mass, *SMI* skeletal muscle index, *T-Chol* total cholesterol. *The values in August 2020 are analyzed for these variables

## Discussion

Following the COVID-19 outbreak, from February 2020 to 6 months later, this study highlighted nutritional deterioration in chronic HD patients, suggesting an association with decreased physical activity.

Cases of pneumonia of unknown etiology were reported in Wuhan, China, and a similar case was confirmed in Japan on January 16, 2020 [13]. The World Health Organization designated COVID-19 as the name of this novel disease in February 2020. From April 7 to May 25, 2020, the Japanese government declared the first state of emergency due to COVID-19; thus, especially from February 2020 to 6 months later, strict infection control and prevention procedures, including restrictions on outings. As a result, human mobility behavior has decreased, with social contact reduced by approximately 70% in Japan’s capital, Tokyo [14, 15]. A decrease in total physical activity times in April 2020 compared to January 2020 was uncovered [3]. After the spread of COVID-19, increased BMI and fat mass have been described, suggesting decreased physical activity in Japanese HD patients [16]. The COVID-19 outbreak in the U.S.A. resulted in reduced physical activity in the general population, particularly among the older people, who took fewer steps and faced an increased risk of falls [17]. According to these reports, the COVID-19 pandemic changed our daily lives drastically globally.

GNRI is associated to CVD and all-cause mortality risk in chronic HD patients [18–20]. In addition, SI and NRI-JH were developed to detect patients with malnutrition with a substantial risk of death based on the DOPPS and nationwide prospective cohort study of the JSDT Renal Data Registry [10, 12]. In this study, these nutritional tools were worsening shortly after the first COVID-19 outbreak.

Regarding body composition, a decreased trend in SLM and SCM and an increased trend in BFM were observed shortly after the first COVID-19 outbreak, suggesting a decline in physical activity. In addition, the decreasing rates in the lower limb from February 2020 to 6 months later were greater than those from February 2019 to 6 months later. It has been reported that low physical activity leads to reduce the skeletal muscle index, especially for the lower limb muscles, and physical activities are associated with lower limb muscle size and strength [21, 22]. For this reason, we consider that the change in behavior patterns during the COVID-19 pandemic notably reduced muscle mass, especially in lower limb [14–17]. Although the SLM change rates were smaller compared to those in lower limb strength, the reason for the SLM increase in upper limb and trunk muscle strength is unknown.

These findings suggest that decreased physical activity shortly after the COVID-19 outbreak may have reduced SLM trends and nutritional deterioration [14–16, 23]. Nutritional deterioration is likely related to anemia and was consistent with our study results [24, 25]. In addition, CRP levels had negative associations. Exercise, or increased physical activity, has been reported to lower CRP levels, and the results of this study suggest a decline in physical activity from February 2020 to 6 months later [26, 27]. Surprisingly, the nutritional impact was significant in patients at the urban facility with higher serum Alb levels. The impact by pandemic may have differed depending on region, original physical and social activity. The serum Alb and T-chol levels of enrolled patients in this study were higher than those in the national survey in Japan [1]; thus, the enrolled patients were relatively healthy based on nutritional index factors. Our results alerted that relatively healthy patients were presumably more affected by the COVID-19 pandemic. Both overnutrition and undernutrition have been uncovered related to the COVID-19 pandemic [4]. However, the patients weren’t overnutrition in this study subjects during the COVID-19 pandemic.

Although there were no significant differences in primary endpoints, post-HD Bw and fluid removal rate reduction were observed immediately after the COVID-19 pandemic. This result contradicts existing reports that Bw gain was observed during the COVID-19 pandemic in the U.S.A. and Japan [16, 28–30]. However, changes in Bw associated with reduced physical activity were also uncovered in the general Japanese population, particularly among the older [31]. Hence, Bw reduction is also associated with decreased physical activity in this study subjects.

The strength of this study was that the magnitude of change in nutritional indicators and body composition was revealed after approximately 2 years of observation. Moreover, the regional characteristics were evaluated. The lessons from the COVID-19 outbreak suggest nutritional status is affected in just several months when restricted daily life by any social crisis including disaster. Consequently, comprehensive support is necessary to ensure the safety and well-being of chronic HD patients when facing these crises.

Some limitations of this study must be noted. First, it is a retrospective study, and as such, patient selection and information biases are present, and a questionnaire survey on physical activity could not be performed. Second, the number of patients enrolled in certain facilities was small, which limits the reliability and certainty of regional differences. Third, in-body tests were conducted in one facility and not on the same day; In addition, measurement times varied before and after HD for each patient. We observed the same seasons before and after the pandemic (August 2019 and 2020, February 2020, 2021, and 2022 making), making it possible to understand seasonal trends. Fourth, the normalized protein catabolic rate and malnutrition inflammation scores could not be evaluated because of missing data. Finally, existing exercise habits may be a confounding factor.

## Conclusion

Shortly after the COVID-19 outbreak, nutritional deterioration was observed in relatively healthy Japanese HD outpatients, suggesting an association with a decline in physical activity.

## Supplementary Information

Below is the link to the electronic supplementary material.

Supplementary file1 (DOCX 14 KB)

## Data Availability

Raw data were generated at Tohoku University, Miyagi, Japan. Derived data supporting the findings of this study are available from the corresponding author on request.
