# Supplementary material for: Influence of infection control for COVID-19 on nutrition in relatively healthy Japanese HD patients: a retrospective observational study
Source: Clin Exp Nephrol. 2025 Feb 28;29(7):961–73. doi: 10.1007/s10157-025-02638-3 (PMC12204872; doi:10.1007/s10157-025-02638-3)
Supplement: Supplementary file 1 — Supplementary file1 (DOCX 14 KB) [file 10157_2025_2638_MOESM1_ESM.docx]

Clinical characteristics, laboratory values, nutritional indicators, and body composition are presented as mean ± standard deviation (SD), median (interquartile range; IQR), or the number of patients (%) based on a test of normality. *Pearson's chi*-*squared test* was used easily in the *analysis* of *contingency tables*. The variance analysis (ANOVA) was used to compare four or five groups, and when significant differences were observed, the Tukey-Kramer method was used for intergroup comparisons. A *P*-value of <0.05 was considered statistically significant. Statistical analysis was performed using the JMP pro17 software (SAS Institute Inc., Cary, NC, USA).
